# Evaluating the Impact of the Diabetes Mellitus Strategy for the National Health System: An Interrupted Time Series Analysis

**DOI:** 10.3390/healthcare9070873

**Published:** 2021-07-12

**Authors:** Marta González-Touya, Rocío Carmona, Antonio Sarría-Santamera

**Affiliations:** 1 Hospital Ramón y Cajal, 28034 Madrid, Spain; mgtouya@salud.madrid.org; 2 Institute of Health Carlos III, 28029 Madrid, Spain; rocio.carmona@isciii.es; 3 Department of Medicine, Nazarbayev University School of Medicine, Nur-Sultan 020000, Kazakhstan

**Keywords:** diabetes mellitus, comorbidity, health plan implementation, public policies evaluation, interrupted time series, predictive and preventive strategies

## Abstract

(1) *Background*: Diabetes mellitus is a significant public health problem. Macrovascular complications (stroke, acute myocardial infarction (AMI) and lower limb amputations (LLAs) represent the leading cause of morbi-mortality in DM. This work aims to evaluate the impact of the approval of the Diabetes Mellitus Strategy of the National Health System (SDM-NHS) on hospitalizations for those macrovascular complications related to DM; (2) *Methods*: Interrupted time series applying segmented regression models (Negative Binomial) adjusted for seasonality to data from hospital discharge records with a primary or secondary diagnosis of DM (code 250 ICD9MC); (3) *Results*: Between 2001 and 2015, there have been 7,302,750 hospital discharges with a primary or secondary diagnosis of DM. After the approval of the SDM-NHS, all the indicators showed a downward trend, modifying the previous trend in the indicators of AMI and LLA. The indicators of stroke and AMI also showed an immediate reduction in their rates; (4) *Conclusions*: After the approval of the SDM-NHS, an improvement has been observed in all the indicators of macrovascular complications of DM evaluated, although it is difficult to establish a causal relationship between the strategy and the effects observed. Interrupted time series is applicable for evaluating the impact of interventions in public health when experimental designs are not possible.

## 1. Introduction

### 1.1. Diabetes: A Major Public Health Problem

Diabetes mellitus (DM) is a disease characterized by elevated plasma glucose levels due to a deficit in insulin production, a failure in its action, or a mixture of both. Its development and evolution results from the combination of genetic and environmental factors. Lifestyles play a significant role both in this incidence as well as in the incidence of complications [1,2]. The prevalence of self-reported DM in Spain is 7.8–13.8% and the incidence is 11.6 new cases per 1000 persons/year [3,4,5,6,7].

Patients with DM present with vascular alterations as a consequence of sustained hyperglycemia. Dysregulation in lipid metabolism, arterial hypertension, atherosclerosis, and other factors, such as age or male sex [8], also contribute to the two to four times increase in the cardiovascular risk of these patients [9] which implies an increased risk of stroke, acute coronary events [10,11,12], and lower limb amputations due to neuropathy and peripheral vascular disease [13]. This excess risk is representative of the fact that DM is associated with a high burden of hospitalizations. In Spain, the hospitalization rates of patients with DM in 2011 were 10.3 discharges per 1000 patients [14]. Macrovascular complications, strokes, coronary events, or amputations represent the leading cause of morbidity and mortality of patients with DM, reduce the quality and length of life of patients with DM, and are responsible for enormous direct and indirect costs.

The incidence of DM is increasing substantially worldwide. Current trends indicate that these rates will only continue to rise: the International Diabetes Federation projects that 592 million people worldwide will have DM by 2035 [15]. The increasing incidence of the disease suggests that their burden will grow in the next few years. One consequence of the growing rates of DM is a considerable economic burden for patients, healthcare systems, and society, both in terms of the direct costs of medical care as well as indirect costs of diminished productivity tied to diabetes-related morbidity and mortality. In 2017, the global expenditure on DM patients aged 18–99 years reached USD 850 billion, and it is expected to rocket up to USD 958 billion by 2045 [16]. The costs of DM in Spain have been estimated to represent 8% of the national healthcare expenditure [17].

Although a large body of evidence has shown the efficacy of several treatments and practices to reduce this burden, a marked variability has been documented in its implementation, indicating a substandard level of DM care currently delivered. This situation has led to the need to develop interventions and policies to improve the care in DM.

DM is a global public health problem, showing an increasing prevalence. As a consequence of inadequate multifactorial management, people with DM remain at significantly higher cardiovascular risk, and cardiovascular disease is a major cause of comorbidity and death among people with DM. Given the large burden that this condition exerts on the healthcare systems, the identification of new strategies to monitor and control DM, to better characterize its complications, and more accurately quantify its prevalence has become a major imperative.

### 1.2. The Strategy for Diabetes Mellitus of the National Health System (SDM-NHS)

The Spanish Ministry of Health (SMoH) approved the Strategy for Diabetes Mellitus of the National Health System (SDM-NHS) in 2006 as part of the Quality Plan for the National Health System, indicating a framework of actions within which the Autonomous Communities can develop and monitor care for patients either with diabetes mellitus (DM) or at risk of suffering from it [18,19].

It was first formulated in 2006, and reviewed in 2012 within the plenary sessions of the Interterritorial Council of the NHS (ICNHS), which is the permanent body of coordination, cooperation, communication, and information among the central and regional public health administrations in Spain. The resolutions from the ICNHS are approved by consensus and materialized through recommendations; therefore, they must be adapted to each respective regional structure and legislation before their implementation. The SDM-NHS consists of six main lines, encompassing prevention, diagnosis, healthcare assistance, complications, gestational DM and training, investigating and innovation in DM. The SDM-NHS also includes a proposal of evaluation and follow up with its specific indicators, as well as general indicators related to prevalence and mortality due to DM. The SDM-NHS main objectives were:

- To reduce the prevalence of overweight and obesity in the general population by promoting breastfeeding and healthier lifestyles.
- To enhance DM screening and early DM diagnosis.
- To improve the management of cardiovascular risk factors in patients with DM and appropriate metabolic control, emphasizing self-care.
- To recognize early complications.
- To reduce DM-related morbidity.
- To avoid maternal and fetal complications providing adequate pregnancy planning and follow up in women with DM.
- To promote gestational DM screening, especially in women at higher risk.
- To encourage and support DM research.

Some Autonomous Communities have subsequently published several documents describing the incorporation of the SDM-NHS to their regional health plans, although this fact does not guarantee its implementation.

### 1.3. Indicators for Assessing DM Quality of Care

Defining appropriate indicators and valid methodologies is necessary for implementing and assessing the impact of quality improvement processes. Indicators are required to reflect credible evidence which enables one to link process measures to relevant clinical outcomes. They must be selected upon justifiable criteria, such as the modifiability of the clinical outcome measures, feasibility of data collection and variability across healthcare settings, so that there can be opportunity for improvement. The set of measures selected has to be comprehensive yet parsimonious.

Several types of indicators have been suggested for evaluating the quality of care for DM, typically being process and outcomes indicators. Major limb amputations, myocardial infarctions or strokes are called “endpoints” of the natural history as well as of the clinical course of the disease [20]. Moreover, information concerning these outcomes can be easily obtained from hospital discharge registries. Although such indicators could not be used to compare individually different healthcare providers, indicators that reflect more distal concepts, i.e., the long-term outcomes for the chronically ill patients, are appropriate for policy purposes and useful for overall health system comparisons, because they represent the end product of aggregated care that cannot be attributed to single providers.

Hyperglycaemia exerts a direct effect on endothelial function and the induction and progression of atherosclerosis, but other factors, such as hyperinsulinemia, insulin resistance, and dyslipidemia, are also involved. These mechanisms are responsible for cardiac and vascular injury and represent common pathways for the development of vascular complications. Coronary heart disease, peripheral artery disease, and cerebrovascular conditions are all common among people with DM, and their prevalence increases with worsening glucose status because of the combined effect of a higher risk of accelerated atherosclerosis and other, more direct lipotoxic and gluco-toxic effects. Concomitant risk factors such as smoking, arterial hypertension, obesity, and dyslipidemia further increase the likelihood of these complications [21]. Any strategy aimed to improve DM care has to take into consideration the diverse comorbidities and complications associated with the incidence and progression of DM, both in terms of the type of interventions as well as in the outcomes proposed to determine its impact.

In line with scientific evidence and international standards, the SDM-NHS proposed to strengthen cardiovascular risk management as a critical component of DM management. DM subjects are affected by numerous cardiovascular complications, and early therapeutic intervention is mandatory, focusing on hyperglycemia, hypertension, and dyslipidemia. Nevertheless, and although the relationship between the nature, frequency, and duration of DM care and the rate of admissions to hospital for those above-mentioned complications is complex and still not well understood, from a public health perspective, to inhibit or delay those complications are of utmost importance [22]. This implies the assessment of long-term indicators, including hospitalization from stroke, myocardial infarctions, or amputations, to evaluate DM quality improvement strategies.

### 1.4. Evaluating Public Health Interventions

Evaluating the impact of public policies, such as the SDM-NHS, is an essential issue for accountability, to characterize the adequacy of the proposals and their achievement of the stated objectives. The usual designs for assessing the efficacy of clinical interventions, such as clinical trials, cannot typically be applied to evaluate the impact of public policies. Evaluating the effectiveness of public policies implies a counterfactual analysis to determine if the observed changes are due to the implementation of those policies: comparing what happened with what would have happened in the absence of the intervention, considering that an experimental design, in these cases, would be unfeasible or unethical [23].

Interrupted time series (ITS) analysis has been proposed as a robust design to evaluate the impact of interventions when their implementation occurs at a concrete moment in time [24,25]. For this, it is necessary to have the observed data of the variable of interest during a period (time series) that includes measurements both before and after the application of the intervention, which is the moment of interruption of the series. The effects of the intervention are assessed by changes in the level and slope of the time series, as well as by the statistical significance of the different model parameters.

### 1.5. Aim of This Study

Although when the strategy was approved in 2006, it included a proposal for its evaluation and although it was revised in 2012, an evaluation of its impact is not available yet. The objective of this work is to carry out an ITS analysis and describe the evolution of the time trend of different indicators related to cardiovascular complications in patients with DM before and after the approval of the strategy in 2006, and thus, to determine the effectiveness of the SDM-NHS.

## 2. Materials and Methods

### 2.1. Data

The population was aged 18 or over with a DM code as the main or secondary diagnosis in the Hospital Discharges Minimum Basic Data Set (CMBD) (ICD9MC up to the 5th digit) maintained by the SMoH and a date of hospital discharge between 1 January 2001 and 31 December 2015.

As the SDM-NHS was approved in 2006, the study period selected was 2001–2015 in order to have sufficient data before and after the intervention. Data after 2015 were not included due to methodological issues related to the change in 2016 of the coding of the CMBD diagnoses. Regarding the geographical scope, the 17 Autonomous Communities of Spain and the 2 Autonomous Cities of Ceuta and Melilla were included.

The annual prevalence of DM was estimated from the National Health Surveys carried out between the years 2001 to 2015, considering as the denominator the resident population in the middle of each period (1 July).

### 2.2. Statistical Analysis

An ITS study was performed with a segmented regression analysis to identify the structural changes of each of the time series and the associated relative risks (RRs) were calculated. This is an observational study that will compare the trends in hospitalizations in DM patients for the following 3 dependent variables (Y_t_) that were selected as indicators to assess the evolution of the vascular complications of DM in 2 periods, before and after the approval of the SDM-NHS:

- Hospital discharge rates of amputations in lower limbs (LLA).
- Hospital discharge rates for stroke.
- Hospital discharge rates for episodes of initial care for acute myocardial infarction (AMI).

The independent variables included were:

- Trend of the series: n_1_. The trend was controlled through a variable in the database that counts along the period: this variable starts on the first month of the series and continues to the end of the series.
- Intervention: *interv*. Binary variable with 2 values: 0 for years before 2007 and 1 for years 2007 and later.
- Interaction between intervention and trend: *n_1_.intervn_1_*. This variable represents interaction between trend and time, centered in the moment of the intervention.

Data were examined to identify possible underlying trends, their stability over time and the existence of seasonality, performing a descriptive pre-strategy and post-strategy analysis specifically for each of the indicators analyzed.

Subsequently, the general model of segmented regression was proposed for a change in slope and level in each result variable, defined by the following formula:

Y_t_ = β_0_ + β_1_∙n_1_ + β_2_ *interv*_t_ + β_3_∙(n_1_∙*interv*_t_)

where β_0_ represents the baseline value when n_1_ = 0; β_1_ represents the change in the dependent variable for each increase of 1 unit in n_1_ (i.e., the trend o slope previous to the intervention); β_2_ represents the change of level in the period of time immediately after the and β_3_ represents the change in trend (slope) after the intervention (i.e., the difference in the slope pre- and post-intervention).

In case of a model with only change in slope, the variable *interv*_t_ was excluded, while for models of change on level, the (n_1_∙*interv*_t_) was excluded. These situations were explored for each of the dependent variables (the 3 previously mentioned indicators).

Autocorrelation, that is, the independence between the observations, was controlled by including seasonality in the models (sines and Fourier cosines) and through the graph of the residuals and the partial autocorrelation function.

In each of the models, over-dispersion was evaluated to determine in each case the use of Poisson or Negative Binomial models [26].

## 3. Results

During the period 2001–2015, 7,302,750 hospital discharges with a diagnosis of DM (either as the main or secondary diagnosis) were registered, of which 54.3% were male. The mean age of the registered discharges was 72 years (SD: 13). Type 2 DM (T2DM) was present in 97.1% of the cases. In-hospital mortality represented 6.8% of total discharges for those causes. Regarding chronic complications of DM, during the period analyzed, 1.96% of hospital discharges registered corresponded to stroke (143,034 records), 3.17% to AMI (231,769 records) and 1.52% were patients who underwent LLA (110,914 records). The main data for each of the years of study are described in Table 1.

Since for all models, the over-dispersion of the different indicators was statistically significant, Negative Binomial models were considered.

### 3.1. Stroke

**Figure 1:**
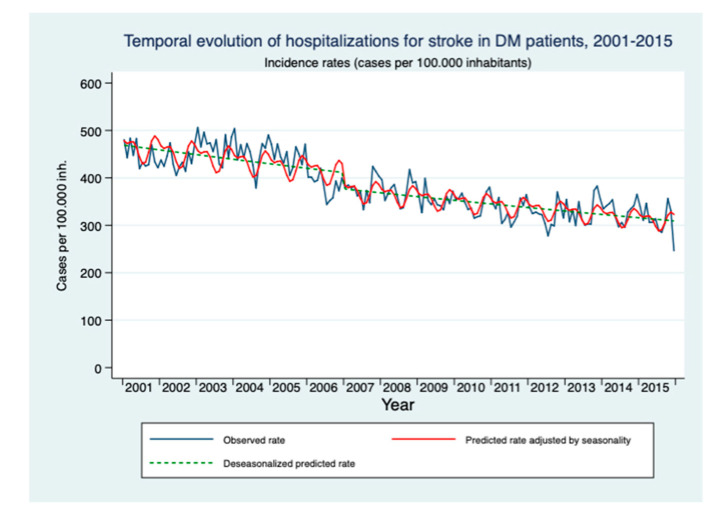
Modeling of the temporal evolution of the stroke hospitalization rate in people over 18 years with DM in the period 2001–2015.

The mean age of patients with DM who were hospitalized with a main diagnosis of stroke was 73 years in the period 2001–2006 and 74 years in the period 2007–2015. The rate of hospitalizations for stroke decreased from 447.16 in 2001 to 311.22 per 100,000 inhabitants/year in 2015. The segmented regression analysis, after adjusting for seasonality, suggests that there was a trend towards reduction prior to the approval of the SDM-NHS, but an immediate reduction of 8.4% (RR = 0.917; 95% CI: 0.886–0.951) is observed in the rate of hospitalizations due to stroke in patients with DM after the strategy was approved (Table 2). Figure 1 shows graphically this reduction.

### 3.2. AMI

**Figure 2:**
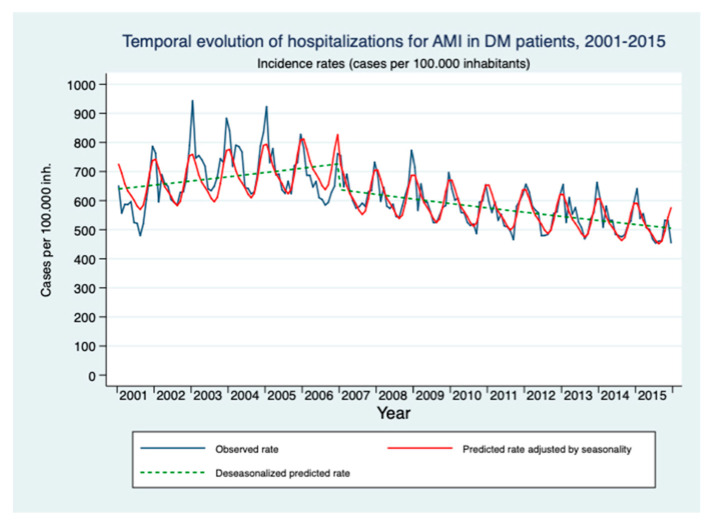
Modeling of the temporal evolution of the hospitalization rate for initial care for an episode of AMI in people over 18 years of age with DM in the period 2001–2015.

The mean age of patients with DM who were hospitalized for initial care for an AMI episode was 71 years in both periods, 2001–2006 and 2007–2015. The rate of hospitalizations for an initial episode of AMI in patients with DM in 2001 was 591.04 admissions per 100,000 inhabitants/year. Before the approval of the strategy, the rates of hospitalization for initial attention to an episode of AMI increased at a rate of 0.2% per month (RR = 1.002; 95% CI: 1.001–1.003). After the approval of the strategy, a 0.4% decrease per month and an immediate reduction of 12.6% (RR = 0.874; 95% CI: 0.839–0.911) could be observed (Table 2 and Figure 2).

### 3.3. LLA

**Figure 3:**
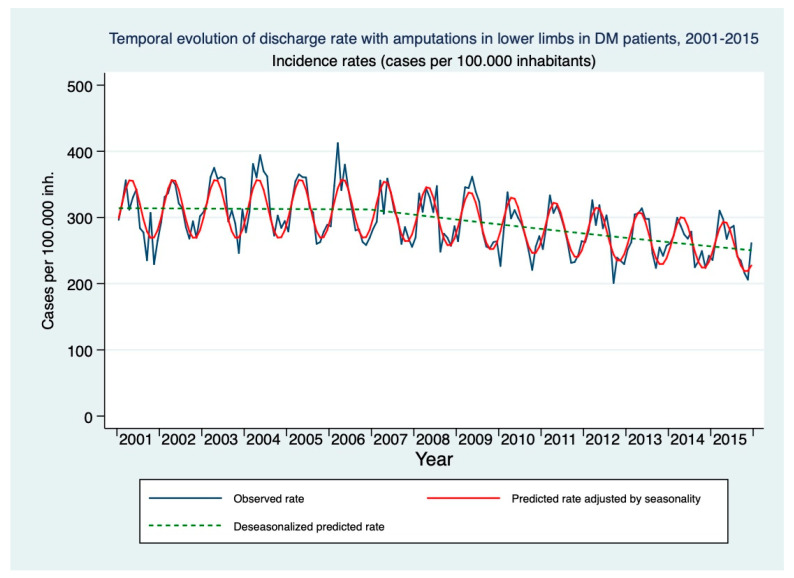
Modeling of the temporal evolution of the rate of hospital discharges with lower limb amputations in people over 18 years of age with DM in the period 2001–2015.

The mean age of DM patients who underwent amputations in lower limbs was 70 years in both the 2001–2006 period and the 2007–2015 period. The most frequent type of amputation in both periods was the amputation of a toe, with 45.5% of cases in the period 2001–2006 and 50.3% in the period 2007–2015, followed by amputation above the knee (30.2% in the first period; 26.5% in the second period). A decrease in the trend of the rates of hospital discharges for lower limb amputations was observed, going from 296.31 amputations per 100,000 inhabitants/year in 2001, reaching 259.07 in 2015 (Figure 3). In the pre-strategy period, the rate of hospital discharges with LLA increased at a monthly rate of 0.002% (RR = 1.00; 95% CI: 0.999–1.001). After its approval, there was a monthly decrease of 0.2% (RR = 0.998; 95% CI: 0.997–0.999) (Table 2 and Figure 3).

## 4. Discussion

### 4.1. Main Findings

The findings of this work, based on the analysis of national health data covering the entire adult population of Spain over 15 years, indicate a substantial and favorable evolution of the incidence of chronic macrovascular complications after the approval of the SDM-NHS. Patterns of these decreases are different for each of the three indicators considered. There was an immediate change in the level of the trend in the stroke hospitalizations rate (previously already decreasing), a change both in the trend as in the level of the same in the rate of hospitalizations for AMI and a change in the trend of LLA, which begins to descend after the approval of the SDM-NHS in the year 2006. Declines in the incidence of macrovascular complications, AMI, stroke or LLA, have also been identified in other countries, in parallel with declining mortality [27,28].

The Saint Vincent Declaration, signed in 1989, established standards of care to be delivered to patients with DM for the first time, as well as programmatic targets addressed to improve the expectancy and quality of life in patients with DM, underlining the importance of reducing complications associated with the disease. Since then, numerous countries have been developing plans and implementing strategies to optimize DM care, in Europe and elsewhere. This work has analyzed 3 of the 52 indicators proposed in the SDM-NHS in Spain. The selection of those indicators was based on their relevance as well as on the availability of data to analyze the trends of macrovascular complications.

Stroke, AMI and LLA hospitalization rates in the diabetic population are important outcome indicators for macrovascular disease. They are considered endpoints that can be influenced by the preventive treatment of macrovascular risk factors [29]. Improving health outcomes is the ultimate goal of the healthcare system. Reducing variability in medical care should represent the main aim of quality measurement initiatives. In the case of DM, it has been demonstrated how the improvement and more homogeneity in the delivery of care have been linked to a substantial reduction in the incidence of cardiovascular events. The use of more standardized approaches could lead to sizable savings in care and outcomes. Outcome measures have been criticized because they can be affected by factors other than the quality of care, but process measures either are absent of limitations, as they alone do not fully describe the whole process of care. Furthermore, positive results in the short run may fail to be sustained in the long run, being unclear to what extent process and intermediate outcome measures predict long-term effects on patients’ health.

### 4.2. Limitations of Observational Data

These results do not allow us to assume, in any case, a causal association between the approval of the SDM-NHS and the improvements observed in the analyzed indicators but permit us to raise hypotheses that might explain the observed effects.

The approval of the SDM-NHS may have generated a dynamic that resulted in the better control of glycaemia levels and the rest of the biochemical blood parameters typically used to monitor and estimate the quality of care for patients with DM as well as associated cardiovascular risk management. The final effect of those changes would have been the improvement of cardiovascular indicators related to DM [30,31]. However, the degree of granularity of the data available does not enable us to ascertain whether improvements in the control of variables such as HbA1c, blood glucose, BP, cholesterol or body mass index have been registered over the period analyzed, nor whether there have been changes in the patterns of treatment of DM. Our study cannot establish neither the impact of the new insulins or oral antidiabetics drugs on DM macrovascular complications hospitalization rates, nor the possible influence of health promotion programs related to diet or physical activity [32]. Furthermore, the improvement observed after the approval of the SDM-NHS in the three DM indicators studied, could be motivated not only by a better glycemic control of DM, but by an intensified cardiovascular management of cardiovascular risk.

On the other hand, the real degree of implementation within each regional health service of the different strategic lines described in the SDM-NHS is unknown. Given the high level of decentralized decision making in Spain, which is a defining characteristic of its national healthcare system, there may have been differences in the specific performance of the interventions proposed in the SDM-NHS in each of the 17 Spanish regions [33]. Different regions may have prioritized certain specific activities over others from those indicated in the SDM-NHS. Additionally, each region may have carried out different approaches in terms of quality assessment methods. The effects those differences may have had on outcomes cannot be estimated because regions do not routinely provide detailed information regarding the specifics of the implementation of the SDM-NHS in their jurisdictions.

### 4.3. Evaluating the Impact of the SDM-NHS

The evaluation of the impact of public policies constitutes an obligation in public health as part of the implicit social responsibility in the management of public resources, although it encounters obvious barriers, not only in the type of methodological design required but also in the collection of reliable data to be evaluated [34].

ITS represents a valuable study design to assess the effectiveness of population-based interventions aimed at quality improvement, and may be particularly useful in public health when randomization is not possible or feasible. One of the criticisms of ITS is that it presents limitations regarding internal validity. Nevertheless, typical threats to internal validity, such as maturation (given that the data were adjusted) or testing, would probably not have any effect in this case. However, the possible influence of events that occur simultaneously in time could not be ruled out [35]. In 2008, Spain was immersed in a deep economic crisis with repercussions at multiple levels, including health, directly or indirectly; the change in some eating patterns with an increase in the consumption of unhealthy but cheaper foods could have been marked by the economic situation [36]. An attempt has been made to control instrumentation by limiting the study period until 2015, the year from which the coding of diagnoses at discharge in the CMBD registry was carried out with the ICD-10 instead of with the ICD-9MC as had been carried out until then.

The evaluation of chronic care represents a significant public health challenge [37]. Accurate measurement at the population level is necessary to inform disease surveillance, public health and intervention strategies, healthcare planning, and research [38]. Administrative databases are increasingly being used for those purposes, because it is a relatively inexpensive data source that provides population-level information over long periods. The validity of these data appears to be higher for well-defined chronic diseases requiring long-term management, such as DM [39]. Countries such as the United Kingdom, Austria or Germany have also relied on observational designs using administrative data to evaluate nation-wide programs to improve quality of diabetes care. Those programs showed significant improvement in quality of care, but the design and data source cannot entirely rule out an influence by residual and unmeasured confounding [40,41].

This work reflects an improvement in the indicators of cardiovascular complications associated with the approval of the SDM-NHS, although neither the information here analyzed nor the design used allows us to causally attribute this improvement with the approval of the SDM-NHS. Other studies with different approaches are necessary for the adequate monitoring of the quality of DM care to ensure the best possible care for people with DM in all phases of the disease.

## 5. Conclusions

The findings of this work reveal a significant improvement after the approval of the SDM-NHS of the three indicators related to macrovascular DM complications analyzed here. ITS analysis has proven to be a suitable instrument for the evaluation of public policies in complex interventions such as the SDM-NHS when experimental studies are not feasible. Nevertheless, the observational nature of this study does not permit the attribution of those reductions to the implementation of the SDM-NHS. More investigation with more granular data may be required to offer adequate healthcare standards all over the course of the disease.

## Figures and Tables

**Table 1:** Distribution of the number of hospital discharges with a primary or secondary diagnosis of DM for each year of study. Count and relative percentage of cardiovascular complications in each year of the study with respect to the total discharges.

Year	Total Hospital Discharges	Women (%)	Mean Age (Years ± SD)	Discharges with Stroke Diagnosis (%)	Discharges with AMI Diagnostic (%)	Discharges with LLA (%)
2001	319,894	49.10%	70 ± 13	9072 (2.84%)	11,856 (3.71%)	6001 (1.88%)
2002	336,368	48.60%	70 ± 13	8961 (2.66%)	13,417 (3.99%)	6373 (1.89%)
2003	383,294	47.90%	70 ± 13	9790 (2.55%)	15,435 (4.03%)	6777 (1.77%)
2004	404,420	47.50%	71 ± 13	9871 (2.44%)	15,857 (3.92%)	7058 (1.75%)
2005	428,470	47.10%	71 ± 13	10,016 (2.34%)	16,176 (3.78%)	7034 (1.64%)
2006	442,867	46.90%	71 ± 13	8930 (2.02%)	15,312 (3.46%)	7321 (1.65%)
2007	474,985	46.30%	71 ± 13	9251 (1.95%)	15,561 (3.28%)	7333 (1.54%)
2008	500,947	46.00%	72 ± 13	9513 (1.9%)	15,652 (3.12%)	7604 (1.52%)
2009	527,659	45.50%	72 ± 13	9421 (1.79%)	15,925 (3.02%)	7968 (1.51%)
2010	543,567	45.10%	72 ± 13	9577 (1.76%)	15,886 (2.92%)	7657 (1.41%)
2011	558,721	44.70%	72 ±13	9560 (1.71%)	15,858 (2.84%)	7963 (1.43%)
2012	571,454	44.40%	73 ± 13	9408 (1.65%)	16,250 (2.84%)	7909 (1.38%)
2013	585,253	44.00%	73 ± 13	9832 (1.68%)	16,545 (2.83%)	8085 (1.38%)
2014	603,186	43.80%	73 ± 13	10,007 (1.66%)	15,989 (2.65%)	7858 (1.3%)
2015	621,665	43.50%	73 ± 13	9825 (1.58%)	16,050 (2.58%)	7973 (1.28%)
Total	7,302,750	45.70%	72 ± 13	143,034 (1.96%)	231,769 (3.17%)	110,914 (1.52%)

**Table 2:** Significant segmented regression models of the indicators related to complications. Relative risks (RRs) and 95% confidence interval (95% CI).

Indicators of Complications in Diabetes Mellitus	RR (95% CI)		
	Stroke	AMI	LLA
Pre-Strategy Trend	0.998 (0.997–0.998)	1.002 (1.001–1.003)	1.000 (0.999–1.001)
Level change after Strategy	0.917 (0.886–0.951)	0.874 (0.839–0.911)	
Trend change after Strategy		0.996 (0.995–0.997)	0.998 (0.997–0.999)
Basal level	0.005 (0.005–0.005)	0.006 (0.006–0.007)	0.003 (0.003–0.003)
All models were seasonally adjusted			
Rates × 100.000 inhabitants			

## Data Availability

Data available on request.
